# Frequency and Associated Factors for Anxiety and Depression in Pregnant Women: A Hospital-Based Cross-Sectional Study

**DOI:** 10.1100/2012/653098

**Published:** 2012-05-02

**Authors:** Niloufer S. Ali, Iqbal S. Azam, Badar S. Ali, Ghurnata Tabbusum, Sana S. Moin

**Affiliations:** ^1^Department of Family Medicine, The Aga Khan University, Stadium Road, P.O. Box 3500, Karachi 74800, Pakistan; ^2^Department of Community Health Sciences, The Aga Khan University, Stadium Road, P.O. Box 3500, Karachi 74800, Pakistan; ^3^Department of Peadiatrics and Child Health, The Aga Khan University, Stadium Road, P.O. Box 3500, Karachi 74800, Pakistan; ^4^Department of Rheumatology, Waikato Hospital, Pembroke Street, Hamilton 3204, New Zealand

## Abstract

Antepartum anxiety and/or depression is a major public health problem globally. The aim of this study was to estimate the frequency of antepartum anxiety and/or depression among pregnant women. This was a cross-sectional study conducted in a tertiary care hospital among pregnant women. A total of 165 pregnant women were interviewed by a clinical psychologist using HADS for assessing anxiety and/or depression and also collected information regarding sociodemographic, obstetric, family relationships, and home environment. Out of the total of 165 pregnant women about 70 percent of them were either anxious and/or depressed. The increasing age of women (*P*-value = 0.073), not having any live birth (*P*-value = 0.036), adverse pregnancy outcome in past including death of a child, stillbirth or abortion (*P*-value = 0.013), participant's role in household decision making (*P*-value = 0.013), and domestic violence (verbal or physical abuse towards mother or children by any family member) (*P*-value = 0.123). Our study highlights that anxiety and/or depression is quite common among pregnant women. Therefore, there is a need to incorporate screening for anxiety and depression in the existing antenatal programs and development of strategies to provide practical support to those identified.

## 1. Introduction

Depression and anxiety during pregnancy is a major public health problem because of their high prevalence [1–3]. The world Health Organization (WHO) estimates that the depressive disorders will be the second leading cause of global disease burden by 2020 [4]. Rates of depressive illness in women of reproductive age group are reported to be twice than those in men [5]. Some women may experience their first depressive episode during pregnancy, whereas others with a history of depression are at increased risk for its recurrence, continuation, or exacerbation [6, 7]. Recently antenatal anxiety has received increased attention with regards to both its impact on infant outcomes and as a risk factor for postnatal depression [8]. Several cohort studies have reported that the antenatal psychiatric morbidity is the strongest risk factor for postnatal depression [9–13]. Secondly, new evidence shows that depression during pregnancy is also associated with adverse child outcomes [14, 15] including premature births, low birth weight, and poor infant growth [16–18].

Studies from developed countries have reported that depression is the most prevalent psychiatric disorder during pregnancy ranging from 10 to 20% [19–22]. Rates of depression especially during the second and third trimesters of pregnancy have been reported substantially [21]. Kim et al. has reported a prevalence of depression of 26% and anxiety of 10% during pregnancy in a low income, ethnically diverse patients from Minnesota [23]. A prevalence of antepartum anxiety symptoms (29%) and antepartum depressive symptoms (18%) were reported from a population-based study in rural Bangladesh [24]. A higher prevalence of antepartum depressive symptoms (33%) was found in a rural subdistrict in the southwest part of Bangladesh [25]. Among South Indian women, the prevalence of depression during the last trimester was found to be around 16% [26]. A study conducted in a rural area of Pakistan has reported that 25% of women suffered from depression during pregnancy [10]. Another study from an urban community in Pakistan found that 18% of pregnant women were anxious and/or depressed [27]. Most of other prior studies of mental health during pregnancy in Pakistan are hospital based [28]. A study from the antenatal clinic of a teaching hospital at Lahore, Pakistan, has reported 34.5% of pregnant women were suffering from anxiety and 25% were suffering from depression [29]. Almost similar results were found from a tertiary care hospital in Karachi, Pakistan [30].

Several studies have revealed that young maternal age [31], lower women's educational level [2], lower couple's income [2], stressful life events [32], and unemployment [32] are associated with antenatal depressive symptoms. Kendler et al. showed that humiliating events that directly devalues an individual in a core role were strongly linked to risk for depressive symptoms [33]. A systematic review has highlighted that life stress, lack of social support, and domestic violence are significantly associated with increased risk of depression during pregnancy [34]. Kazi et al. has reported that increasing age, lower educational levels, issues regarding husband abuse, extramarital affairs, not giving time to family and putting restrictions on the women and interference by in-laws, and heavy household works were significantly associated with depression during pregnancy [35]. The predictors of antepartum depression and anxiety in an urban community in Pakistan were husband's unemployment, low household wealth, having 10 or more years of formal education, unwanted pregnancy, and partner violence [27]. Partner violence, unsupportive husband and/or mother-in-law, and family preference for son were the predictors of antepartum depression among rural Bangladeshi women [25].

The association between poverty and mental disorder has been elucidated in a review of studies from six low- and middle-income countries [36]. A recent study from an urban community in Pakistan has also found a positive association between lower household wealth and antepartum anxiety/depression [27]. A multicentre prospective study of perinatal depression in Japan reported poor accommodation (rented accommodation, dissatisfaction about accommodation) to be a risk factor for antenatal depression [37]. Literate women are more likely to have good social networks and social support which has been identified as a protective factor in previous research studies [38–40]. In contrast, a US-based study has highlighted education as a risk factor [41].

Despite the high prevalence of depression and anxiety during pregnancy and their significant negative impact, this is still relatively less explored area in Pakistan. The aim of the study was to estimate the frequency and associated risk factors for depression and anxiety in pregnant women.

## 2. Material and Methods

A cross-sectional study was conducted in pregnant women attending antenatal clinics of The Aga Khan University Hospital in Karachi, Pakistan, for their routine antenatal checkups. Women who consented to participate in the study were interviewed using a precoded structured questionnaire comprising of sociodemographic, home environment, and family relationships variables followed by hospital anxiety depression scale (HADS) to assess the current status for anxiety and depression among participants. A total of 165 pregnant women were interviewed from September 2005 till January 2006.

### 2.1. Instrument Used

#### 2.1.1. Hospital Anxiety and Depression Scale (HADS)

HADS is a commonly used instrument in hospital setting to determine anxiety and/or depression. It is a ten-point scale used to determine anxiety or depression separately. A total score of ≥8 on the depression or anxiety scale was taken as positive for anxiety or depression. Anxiety or depression status was taken as the outcome variable in the study. Using this instrument, study participants were classified as normal, only anxious, only depressed, or both anxious and depressed. Validated HADS in Urdu language (lingua franca) was used for interviewing our participants [42]. The Urdu translated version of HADS demonstrated satisfactory linguistic equivalence, conceptual equivalence, and scale equivalence (concordance rates at the cutoff of 8 for anxiety and depression subscales were 82.4% and 87.0%, resp., and at the cutoffs of 11 were 91.7% and 98.1%, resp.) with English version [42].

#### 2.1.2. Sociodemographic, Obstetric, Family Relationships, and Home Environment Questionnaire

We designed a precoded structured questionnaire in English after extensive literature search. The questionnaire included woman's age, ethnicity, education, working status, husband occupation, number of household members, total pregnancies, total live births, total abortions, total stillbirths, total children died, reasons for their deaths, willingness for current pregnancy, ever used any family planning method, past history of any self/family psychiatric disorders, family support, and physical or mental violence by immediate partner or any other family members. The questionnaire was translated into Urdu and then back-translated in English. The Urdu version of the questionnaire was used for interviewing the participants.

### 2.2. Data Collection

Data was collected by a clinical psychologist who filled out the precoded structured questionnaire comprising of sociodemographic, obstetric, family relationships, and home environment characteristics along with HADS from the study participants.

### 2.3. Statistical Analysis

A 95% confidence interval for anxiety and/or depression was calculated using no anxiety and/or depression as reference category (binary). Pie chart was created to show all four categories of anxiety and/or depression. Mean and standard deviation for variables like age of the respondent were also computed. Cross tabulations between the sociodemographic variables and the four categories of anxiety and/or depression were generated. Crude associations between these factors were assessed using simple multinomial logistic regression. Factors were included into the multivariable analysis using multinomial logistic regression, if they were associated with the outcome with *P* value < 0.25 at univariate analysis. Multicollinearity between independent factors was also examined using phi test. The strength of associations between independent factors and anxiety and/or depression (outcome) using no anxiety and/or depression as reference category for univariate and multivariable analyses was reported as crude and adjusted odds ratios with 95% confidence intervals. SPSS (statistical package for social sciences) for Windows (version 19.0) software was used to analyze the data.

### 2.4. Ethical Consideration

The study was approved by the Family Medicine's Departmental Research Committee of the Aga Khan University Hospital, Karachi, Pakistan.

## 3. Results

**Figure 1:**
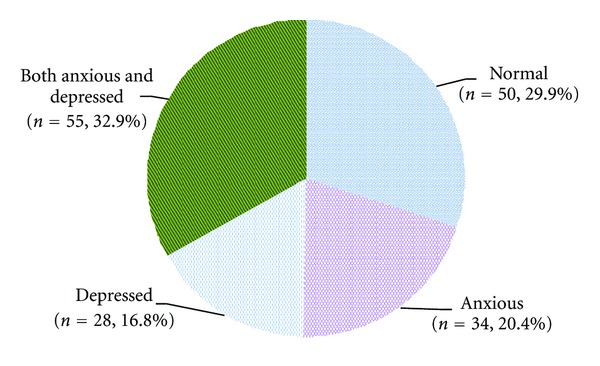
Distribution of study participants by their anxiety and depression status (*n* = 167).

A total of 167 pregnant women were enrolled in the study. About seventy percent of them were either anxious or depressed or both (70.1%; 95% CI: 63.1, 77.0). Most of these women were both anxious and depressed (Figure 1).

Mean age of study participants was 27.92 years (standard deviation = 4.7 years). Majority of the women were housewives (76.6%). More than three-fourth of the participants were speakers of Urdu as their native language (78.4%), and more than two-thirds were graduate or above (70%). Median number of persons living in a household were five (ranged: 2 to 15 persons per household), and median number of pregnancies among the study participants was two (ranged: one pregnancy to 7 pregnancies). More than half of them were having at least one live birth (55.7%). Approximately 95% of the women did not report any history of child death. Only 2 participants reported having pregnancies resulting in still births. Majority of the women reported a willingness to have the pregnancy (91%). Twenty-eight percent women reported having ever used family planning methods, while about half of them had intention of using any family planning method (52.7%). A quarter of the women reported that they could decide to use family planning method themselves (25.7%), and a similar proportion reported family planning method use in consultation with their husbands (27.5%). About 15 percent of them reported psychiatric treatment for themselves or any member in the family. More than ninety percent of the study participants were satisfied with their lives. About one-fourth of the study participants were worried about their household environment. Approximately 86% of the participants reported seeking help to reduce worry. Majority of them were involved in household decision making. Most of them reported domestic violence (physical or mental). The distributions of different characteristics of mothers with anxiety and depression status are given in Table 1.

The univariate analysis for anxiety and depression status is provided in Table 2. Age of the study participant (*P* value = 0.049), total number of live births (*P* value = 0.018), respondent's involvement in household decision making (*P* value = 0.018), and adverse pregnancy outcome (including any death of child, abortion or stillbirth) (*P* value = 0.037) were found to be significantly related to anxiety and depression. Other important characteristics were ethnic background of the respondents (*P* value = 0.187), total number of pregnancies (*P* value = 0.152), domestic violence (verbal or physical abuse towards mother or children by any family member) (*P* value = 0.19), and total number of persons living in the household (*P* value = 0.295) were considered for inclusion in multivariable analysis. Satisfaction with life (*P* value = 0.001) although found to be significant was not included in the multivariable analysis because all the women who were not satisfied with their lives were found to be either anxious, depressed, or both and resulted in distorting the model. No significant difference was observed for respondent's educational status, women's working status, willingness of pregnancy, ever used family planning methods, intention to use family planning methods, could decide to use family planning method themselves, psychiatric treatment for themselves or any member in the family, worried about household environment, and sought help for the reduction of worry.

The multivariable analysis for anxiety and depression status is provided in Table 3. The variables included in the model were age of women (*P* value = 0.073), total live births (*P* value = 0.036), adverse pregnancy outcome (*P* value = 0.013), respondent's role in the household decision making (*P* value = 0.013), and domestic violence (verbal or physical abuse towards mother or children by any family member) (*P* value = 0.123).

## 4. Discussion

In our study nearly 70% of the screened pregnant women were either anxious, depressed, or both. Almost similar findings were observed from Lahore, Pakistan, [29] and Hong Kong [43], where studies were conducted in hospital settings and had also used HADS as an instrument for measuring anxiety and depression. Hamirani et al. [30] from Karachi, Pakistan, has reported frequency of antenatal depression of 34.6% using Edinburgh postnatal scale. Niaz et al. [29] has found lower rates by using ICD-10 diagnostic criteria for measuring anxiety and depression as compared to HADS on the same patients. The probable reasons for this difference could be that ICD-10 system has restrictive definitions as compared to HADS and also HADS is a self-administered instrument. The rates vary depending upon the types of instrument used [44] for measuring antenatal anxiety and depression.

Sociodemographic and psychiatric correlates of anxiety or depression in nonpregnant women are well known but not much have been described in pregnant women. In our study increasing age of women, not having any live birth, adverse pregnancy outcome in past, not being involved in decision making of family matters, and domestic violence were associated with either anxiety or depression.

Increasing age was also reported as an associated factor for anxiety and depression among pregnant women [35] as well as in reproductive age group [45]. Some studies have shown that adverse pregnancy outcomes like higher rates of mortality in the offspring and preterm birth are associated with depression during pregnancy [18, 46–48].

Social relations including involvement in household decision making were also found to be significant factor in previous studies [24, 35]. Lack of social support was found to be as a risk factor in various other studies [49–51]. The importance of social relations with husband and in-laws has been found in other cultures as well [52].

Domestic violence in the form of sexual/physical as well as verbal abuse was strongly associated with antepartum depression/anxiety from a recent study from urban community in Pakistan [27]. Gender-based violence has been described as the most important predictor of depression and anxiety in women. This evidence is well documented in high-income countries [53] and is growing in low-income countries [24, 25, 27, 54].

The main strength of our study was to report the frequency of anxiety and depression as none, anxious only, depressed only, and both anxious and depressed with their associated factors, secondly, the use of clinical psychologist for the collection of data including hospital anxiety and depression scale (HADS).

Limitations included hospital-based study in which prevalence cannot be determined, cross-sectional study design in which cause and effect relationship cannot be built between pregnancy and anxiety and depression status, no predetermined sample size as the study was conducted in a prespecified time, self-reported nature of the responses which might be a reason for high frequency of anxiety and depression and for other variables like satisfaction with current life, noninclusion of some important variables like heavy household work, and pregnancy symptoms.

Our study highlights that anxiety and depression are common during pregnancy. Therefore, there is a need to incorporate screening for anxiety and depression in antenatal programs and providing practical support to women during pregnancy, particularly those with a previous history of depression and who have poor family relationship. The study indicates the necessity of integrating mental health with existing maternal and child health program to ensure the health of both mother and baby.

## Figures and Tables

**Table 1:** Frequency of anxiety and depression by sociodemographic, obstetric, family relationships, and home environment characteristics.

Factor		Anxiety or depression status				All
		Normal	Only anxious	Only depressed	Both anxious and depressed	
Age group (in years)						
Below 30	*n*	40	26	20	31	117
	%	34.2%	22.2%	17.1%	26.5%	70.1%
30 and above	*n*	10	8	8	24	50
	%	20.0%	16.0%	16.0%	48.0%	29.9%

Ethnicity						
Urdu	*n*	38	31	21	41	131
	%	29.0%	23.7%	16.0%	31.3%	78.4%
Other	*n*	12	3	7	14	36
	%	33.3%	8.3%	19.4%	38.9%	21.6%

Level of education						
Up to intermediate	*n*	16	10	8	16	50
	%	32.0%	20.0%	16.0%	32.0%	29.9%
Graduate and above	*n*	34	24	20	39	117
	%	29.1%	20.5%	17.1%	33.3%	70.1%

Woman working status						
Working	*n*	12	10	4	13	39
	%	30.8%	25.6%	10.3%	33.3%	23.4%
Housewife	*n*	38	24	24	42	128
	%	29.7%	18.8%	18.8%	32.8%	76.6%

Number of people living in the house						
Up to 5	*n*	32	17	12	31	92
	%	34.8%	18.5%	13.0%	33.7%	55.1%
More than 5	*n*	18	17	16	24	75
	%	24.0%	22.7%	21.3%	32.0%	44.9%

Total pregnancies						
Below 3	*n*	32	26	18	29	105
	%	30.5%	24.8%	17.1%	27.6%	62.9%
3 and above	*n*	18	8	10	26	62
	%	29.0%	12.9%	16.1%	41.9%	37.1%

Total live births						
None	*n*	25	20	6	23	74
	%	33.8%	27.0%	8.1%	31.1%	44.3%
At least one	*n*	25	14	22	32	93
	%	26.9%	15.1%	23.7%	34.4%	55.7%

Adverse pregnancy outcome						
No	*n*	40	22	22	31	115
	%	34.8%	19.1%	19.1%	27.0%	68.9%
Yes	*n*	10	12	6	24	52
	%	19.2%	23.1%	11.5%	46.2%	31.1%

Willingness of pregnancy						
Yes	*n*	45	31	26	50	152
	%	29.6%	20.4%	17.1%	32.9%	91.0%
No	*n*	5	3	2	5	15
	%	33.3%	20.0%	13.3%	33.3%	9.0%
Ever used family planning methods						
Yes	*n*	14	8	10	15	47
	%	29.8%	17.0%	21.3%	31.9%	28.1%
No	*n*	36	26	18	40	120
	%	30.0%	21.7%	15.0%	33.3%	71.9%

Intention of using FP methods						
Yes	*n*	26	20	14	28	88
	%	29.5%	22.7%	15.9%	31.8%	52.7%
No	*n*	20	8	8	21	57
	%	35.1%	14.0%	14.0%	36.8%	34.1%
Have not planned yet	*n*	4	6	6	6	22
	%	18.2%	27.3%	27.3%	27.3%	13.2%

Could decide about FP method use						
Yes	*n*	13	9	9	12	43
	%	30.2%	20.9%	20.9%	27.9%	25.7%
No	*n*	26	13	10	29	78
	%	33.3%	16.7%	12.8%	37.2%	46.7%
Both will take decision	*n*	11	12	9	14	46
	%	23.9%	26.1%	19.6%	30.4%	27.5%

Ever self-treated or any family member for psychiatric disorder						
No	*n*	45	30	24	43	142
	%	31.7%	21.1%	16.9%	30.3%	85.0%
Yes	*n*	5	4	4	12	25
	%	20.0%	16.0%	16.0%	48.0%	15.0%

Satisfied with life						
Yes	*n*	50	31	27	44	152
	%	32.9%	20.4%	17.8%	28.9%	91.0%
No	*n*	0	3	1	11	15
	%	.0%	20.0%	6.7%	73.3%	9.0%

Worried about household environment						
Yes	*n*	10	8	7	19	44
	%	22.7%	18.2%	15.9%	43.2%	26.3%
No	*n*	40	26	21	36	123
	%	32.5%	21.1%	17.1%	29.3%	73.7%

Ever seek help for reducing worry						
Yes	*n*	45	30	22	47	144
	%	31.3%	20.8%	15.3%	32.6%	86.2%
No	*n*	5	4	6	8	23
	%	21.7%	17.4%	26.1%	34.8%	13.8%
Household decision maker						
Self or husband	*n*	19	8	14	18	59
	%	32.2%	13.6%	23.7%	30.5%	35.3%
In laws	*n*	7	12	10	15	44
	%	15.9%	27.3%	22.7%	34.1%	26.3%
Combined	*n*	24	14	4	22	64
	%	37.5%	21.9%	6.3%	34.4%	38.3%

Domestic violence						
Yes	*n*	43	29	21	39	132
	%	32.6%	22.0%	15.9%	29.5%	79.0%
No	*n*	7	5	7	16	35
	%	20.0%	14.3%	20.0%	45.7%	21.0%

**Table 2:** Crude odds ratio (95% CI) by sociodemographic, obstetric, family relationships, and home environment characteristics.

Factor	Anxiety or depression status			
	Normal	Only anxious	Only depressed	Both anxious and depressed
Age group (in years)				
Below 30 (ref.)	Reference	1	1	1
30 and above		1.23 (0.43, 3.53)	1.60 (0.55, 4.68)	3.10 (1.29, 7.42)

Ethnicity				
Urdu (ref.)	Reference	1	1	1
Other		0.31 (0.08, 1.18)	1.06 (0.36, 3.09)	1.08 (0.44, 2.63)

Total persons in the household				
Up to 5 (ref.)	Reference	1	1	1
More than 5		1.78 (0.73, 4.31)	2.37 (0.92, 6.10)	1.38 (0.63, 3.02)

Total pregnancies				
Below 3 (ref.)	Reference	1	1	1
3 and above		0.55 (0.21, 1.46)	0.99 (0.38, 2.59)	1.59 (0.73, 3.49)

Total Live births				
None	Reference	1.43 (0.59, 3.44)	0.27 (0.10, 0.79)	0.72 (0.33, 1.55)
At least one (ref.)		1	1	1

Adverse pregnancy outcome				
No (ref.)	Reference	1	1	1
Yes		2.18 (0.81, 5.86)	1.09 (0.35, 3.40)	3.10 (1.29, 7.42)

Household decision maker				
Self or husband (ref.)	Reference	1	1	1
In laws		4.07 (1.17, 14.15)	1.94 (0.59, 6.36)	2.26 (0.75, 6.83)
Combined		1.39 (0.48, 3.99)	0.23 (0.06, 0.80)	0.97 (0.41, 2.30)

Domestic violence				
Yes	Reference	1.06 (0.31, 3.66)	2.05 (0.64, 6.60)	2.52 (0.94, 6.77)
No (ref.)		1	1	1

**Table 3:** Adjusted odds ratio (95% CI) by sociodemographic, obstetric, family relationships, and home environment characteristics.

Factor	Anxiety or depression status			
	Normal	Only anxious	Only depressed	Both anxious and depressed
Age group (in years)				
Below 30 (ref.)	Reference	1	1	1
30 and above		1.55 (0.45, 5.33)	1.17 (0.35, 3.92)	3.54 (1.24, 10.12)

Total live births				
None	Reference	1.82 (0.64, 5.14)	0.33 (0.10, 1.08)	1.49 (0.57, 3.88)
At least one (ref.)		1	1	1

Adverse pregnancy outcome				
No (ref.)	Reference	1	1	1
Yes		3.25 (1.09, 9.70)	0.92 (0.27, 3.14)	3.57 (1.35, 9.45)

Household decision maker				
Self or Husband (ref.)	Reference	1	1	1
In-laws		5.80 (1.53, 21.92)	2.26 (0.64, 7.93)	3.81 (1.13, 12.83)
Combined		1.64 (0.53, 5.07)	0.28 (0.08, 1.04)	1.36 (0.51, 3.59)

Domestic violence				
Yes	Reference	1.17 (0.32, 4.24)	1.64 (0.48, 5.65)	3.14 (1.10, 8.98)
No (ref.)		1	1	1
